# *Dianthus superbus* L. (QM) Extract-Assisted Silver Nanoparticle Gelatin Films with Antioxidant and Antimicrobial Properties for Fresh Fruit Preservation

**DOI:** 10.3390/foods14132327

**Published:** 2025-06-30

**Authors:** Chenwei Zhang, Yao Li, Yue Huo, Hongtao Wang, Dandan Wang

**Affiliations:** 1 College of Life Sciences, Yantai University, Yantai 264005, China; zchenwei0620@163.com (C.Z.); liyao723@163.com (Y.L.); ytuwht@163.com (H.W.); 2 School of Biological Science and Food Engineering, Chuzhou University, Chuzhou 239000, China; huoyue0214@163.com

**Keywords:** *Dianthus superbus* L. (QM) extracts, AgNPs, green synthesis, antibacterial activity, cytotoxicity, fruit preservation

## Abstract

We synthesized QM-AgNPs (*Dianthus superbus* L.-AgNPs, Qu Mai-AgNPs) by an economical and environmentally friendly method using *Dianthus superbus* L. extract as a reducing and stabilizing agent. The resulting QM-AgNPs were comprehensively characterized and evaluated for their antioxidant, cytotoxic, and antibacterial activities. Herein, TEM analysis revealed that the QM-AgNPs were predominantly spherical, polydisperse, and exhibited a core particle size ranging from 11 to 18 nm. In contrast, DLS analysis showed a larger hydrodynamic diameter (primarily 60–87 nm), reflecting the hydrated shell and surface biomolecular corona. The crystalline nature of QM-AgNPs was confirmed by XRD and SAED spectra while FTIR spectroscopy indicated the presence of functional groups from the plant extract that may contribute to nanoparticle stabilization. Functional assessments demonstrated that QM-AgNPs exhibited strong antioxidant activity, with efficient DPPH radical scavenging, and selective cytotoxicity against A549 cancer cells while sparing normal cells. Moreover, QM-AgNPs showed significant antibacterial activity against both *Staphylococcus aureus* (Gram-positive) and *Escherichia coli* (Gram-negative), likely due to membrane disruption and the leakage of intracellular contents. To explore practical applications, we developed a GEL@AgNPs coating system for the postharvest preservation of grapes. As a result, the reduced weight loss and decay rate suggest a potential role for QM-AgNPs in extending fruit freshness. Comprehensive shelf-life studies are planned to further substantiate the potential of QM-AgNPs as an effective material for active food packaging applications.

## 1. Introduction

Nanomaterials are extensively utilized in fields such as biotechnology, biomedicine, optoelectronics, pharmaceuticals, cosmetics, food industries, materials science, and agriculture [1,2,3,4]. Silver nanoparticles (AgNPs) have particle sizes ranging from 1 to 100 nm [5]. Compared with other nanoparticles, AgNPs have diverse biocompatibility and metal properties; these characteristics have led to widespread research and numerous applications of AgNPs in nanotechnology. Due to their antibacterial properties, AgNPs are frequently used as preservatives in the food industry [6]. They also serve as antioxidants and antibacterial agents in the cosmetics industry, as well as antibacterial agents and catalysts for chemical waste degradation in sewage treatment [7,8,9]. Additionally, AgNPs have unique physicochemical properties that enable specific targeting of tumor cells, without inducing genotoxic effects on human cells at low concentrations [10], thus laying the foundation for novel research in cancer treatment and drug delivery [11].

The main methods for synthesizing AgNPs include physical, chemical, and biological methods [12]. The particle size, morphology, and stability of AgNPs vary depending on the synthesis method used. With growing concerns over the environmental and health impacts of physical and chemical synthesis methods, green synthesis has gained considerable attention. In contrast, biosynthesis methods involve the use of extracts of biological organisms as reducing agents and stabilizers. No toxic substances remain in the product, as no toxic solvents are used [13]. The biological synthesis of silver nanoparticles frequently utilizes bacteria, fungi, yeasts, algae, and plants [14]. Bacterial synthesis of silver nanoparticles can be classified into intracellular and extracellular synthesis. In intracellular synthesis, silver ions penetrate the bacterial cell membrane and enter the cell, where they are reduced to silver atoms by enzymes or metabolic products, subsequently aggregating to form nanoparticles. Extracellular synthesis involves the adsorption of silver ions by extracellular polymers secreted by bacteria, which are then reduced to silver nanoparticles through enzymatic reactions or redox reactions, with the particles typically dispersed in the culture medium. Fungal synthesis of silver nanoparticles occurs through the adsorption of silver ions by functional groups on the fungal hyphal surface, followed by the reduction to silver nanoparticles via intracellular enzymes or extracellularly secreted organic acids, amino acids, and other compounds [15]. Plant-based synthesis methods leverage the active substances in plant extracts as reducing and capping agents, facilitating synthesis with naturally occurring compounds. Numerous studies have demonstrated the successful synthesis of silver nanoparticles from various plant extracts, with variations in size, shape, and surface functional molecules, resulting in diverse biological activities [16,17]. This method can promote waste utilization [10], and make the continued utilization of plant materials for the biosynthesis of AgNPs a prominent area in the recent research.

*Dianthus superbus* L. (QM) is a perennial plant of the Caryophyllaceae family [18]. As a plant with dual medicinal and edible value in TCM, *Dianthus superbus* L. is traditionally recognized for its ethnopharmacological properties, including anti-inflammatory effects (‘clearing heat’), diuretic activity, and historical use in regulating blood circulation-related menstrual disorders (‘breaking blood through menstruation’) [19]. Additionally, QM extracts exhibit inhibitory effects on *Escherichia coli* and *Staphylococcus aureus* [13,20]. Therefore, we utilized QM as the raw material for preparing AgNPs, the synthesis method of which is green and relatively simple. Furthermore, the high moisture content in fruits leads to a short shelf life and quality degradation during transportation, causing browning, rotting, and other issues [21]. Previous studies [22,23,24,25] have demonstrated that composite membranes prepared by combining AgNPs with other substances possess antibacterial effects.

Our study utilized the QM extract as a stabilizing and reducing agent to optimize the synthesis conditions for AgNPs. Cytotoxicity assays were performed to demonstrate the inhibitory effects of synthesized QM-AgNPs on A549 and Human Umbilical Vein Endothelial Cells (HUVECs). The bacteriostatic mechanisms, including zone of inhibition, minimum inhibitory concentration (MIC), minimum bactericidal concentration (MBC), growth dynamics curve, membrane permeability, reactive oxygen species (ROS), and antibiofilm activity, were conducted to confirm the bacteriostatic effects of QM-AgNPs against *E. coli* (ATCC 25922) and *S. aureus* (ATCC 29213). Finally, grapes were used to test the fruit preservation capabilities of QM-AgNPs, which confirmed the effect of QM-AgNPs in extending the shelf life of fruit [26], and provide certain reference values for the development of the fruit preservation industry [27].

## 2. Materials and Methods

### 2.1. Chemicals and Materials

Silver nitrate was purchased from Sinopharm Chemical Reagent Co., Ltd. (Shanghai, China), for use. All reagents used in our study were of analytical grade. *Dianthus superbus* L. roots were obtained from Yantai Herbal Market. LB broth medium (LB) and agar were provided by the laboratory of Yantai University. *E. coli* (ATCC 25922) and *S. aureus* (ATCC 29213) were obtained from the laboratory of Yantai University. The A549 and HUVECs were obtained from the Shanghai Cell Bank of the Chinese Academy of Sciences. The fruits used in the freshness preservation experiment were purchased from the fruit store at Yantai University.

### 2.2. Preparation of the QM Extraction

A total of 10 g QM powder was placed in a conical flask with 100 mL of deionized water (DDW) and heated in a water bath at 90 °C for 100 min. Moreover, the extracts were centrifuged at 10,611× *g* for 10 min using a high-speed centrifuge, then the supernatant was transferred to a refrigerator at 4 °C for storage and future use.

### 2.3. Green Synthesis of AgNPs

AgNO_3_ (1 mol/L) and QM extract were added to the conical flask (200 mL extract: 200 μL AgNO_3_). After thorough mixing, the reaction mixture was placed in a 90 °C water bath for 60 min. During the reaction, a color change occurred (from light yellow to brown black), indicating the synthesis of silver nanoparticles [28]. After the reaction, the mixture was centrifuged at 10,611× *g* for 10 min, and then washed repeatedly by centrifuging at 12,000 rpm for 15 min. The precipitate was dried at room temperature for subsequent use.

### 2.4. Optimization of QM-AgNP Synthesis Conditions

#### 2.4.1. Single-Factor Design

To optimize the conditions for the synthesis of QM-AgNPs, we employed a single-factor design focusing on extract concentration, synthesis time, and reaction temperature. The optimal conditions for these three factors were determined by measuring the absorbance values.

#### 2.4.2. Box–Behnken Design for Optimization

To optimize the synthesis conditions for silver nanoparticles, we focused on three variables: extract concentration, reaction time, and reaction temperature. Gradients for each variable were established, and experimental protocols were designed using Design-Expert 13 to optimize the synthesis conditions [29], as shown in Table 1. After the reaction, the mixtures were scanned using a dual-beam UV-visible spectrophotometer, and the resulting absorbance values were analyzed with the software. Finally, the optimized conditions were validated through experiments.

### 2.5. Characterization of QM-AgNPs

AgNPs were analyzed by UV-Vis spectrophotometry within the wavelength range of 300~700 nm. Moreover, the size, morphology, and crystal structure of the prepared AgNPs were characterized using transmission electron microscopy (TEM). The elemental composition of the sample microstructure was analyzed using energy-dispersive X-ray spectroscopy (EDX) [30]. The crystal structure of AgNPs was determined by X-ray diffraction [31] (XRD) and selected area electron diffraction (SAED). Furthermore, the particle size of QM-AgNPs was measured by dynamic light scattering (DLS) [32]. Attenuated total reflectance Fourier-transform infrared (ATR-FTIR) spectroscopy was used to obtain molecular bond information, with spectra recorded in the range of 500–4000 cm^−1^ using a diamond probe.

### 2.6. Antioxidant Activity of QM-AgNPs

Different concentrations of the QM-AgNP solution were added to each tube, with a 2,2-Diphenyl-1-picrylhydrazyl (DPPH) solution serving as the negative control group and vitamin C (Vc) as the standard control group. Furthermore, 2 mL of DPPH–ethanol solution (0.10 mmol/L) was added to each tube, and the mixture was vortexed to ensure even mixing. The reaction was allowed to proceed in the dark for 30 min, and the absorbance of each sample was measured at 517 nm. The formula for calculating the DPPH radical scavenging rate is as follows:

DPPH radical scavenging rate (%) = [1 − (As − A_0_)/A_0_] × 100%

In the formula, A_0_ represents the absorbance value of the DPPH–ethanol solution; As represents the absorbance value after the addition of QM-AgNPs; and Ac represents the absorbance value of the QM-AgNPs solution alone.

### 2.7. In Vitro Cytotoxicity

#### 2.7.1. Cell Viability

A549 cancer cells and HUVECs were cultured in Dulbecco’s Modified Eagle Medium (DMEM) and RPMI-1640 medium supplemented with 10% fetal bovine serum (FBS) at 37 °C in a humidified atmosphere containing 5% CO_2_ and 95% air. The cytotoxicity of QM-AgNPs in A549 and HUVECs was determined using the the Methylthiazolyldiphenyl-tetrazolium bromide (MTT) assay.

#### 2.7.2. Cell Apoptosis Detection

The slides were sterilized by soaking them in an ethanol solution and then rinsed repeatedly with sterile Phosphate Buffered Saline (PBS). Cells with a density of 1 × 10^5^ were inoculated into six-well plates containing the cell culture slides and placed in an environment of 37 °C, 5% CO_2_ for 24 h. The next day, different concentrations of QM-AgNPs were added to the wells to treat lung cancer cells for 24 h. After 24 h of incubation, the previous culture medium was discarded, and a fixative was added to fix the cells for 20 min. After washing with PBS for 3 min, Hoechst staining solution was added and the cells were stained for 5 min at room temperature and protected from light. The staining solution was discarded and rinsed with PBS. A drop of anti-fluorescence quenching agent was added to the crawler sheet with a pipette, the culture slides with cells attached were covered, and the morphology was then observed and photographed under a fluorescence microscope.

### 2.8. Antibacterial Properties of QM-AgNPs

#### 2.8.1. Determination of the Inhibition Zone

The paper-disk diffusion method [33] was employed to observe the sensitivity of Gram-negative bacteria *E. coli*, as well as Gram-positive bacteria *S. aureus* to QM-AgNPs and amikacin (positive control).

#### 2.8.2. Minimum Inhibitory Concentration (MIC)

The MIC was determined using the microdilution method [34]. The activated bacterial suspension was diluted to 1 × 10^6^ CFU/mL. Liquid medium, QM-AgNPs at concentrations ranging from 4 to 512 μg/mL, and a bacterial solution were added to the 96-well plate, ensuring that the final volume of each well was 200 μL. All samples were incubated at 37 °C for 24 h. On the following day, MTT solution (5 mg/mL) was added and then incubated for 4 h. The color of the reaction solution in the 96-well plate was observed, and the lowest concentration that did not change color was recorded as the MIC.

#### 2.8.3. Minimum Bactericidal Concentration (MBC)

The bacterial solutions containing different concentrations of QM-AgNPs were pipetted onto the plates and spread evenly. The plates were then incubated at 37 °C for 16–20 h. The concentration was recorded as MBC when no colony growth or less than 5 colonies were observed on the plate.

#### 2.8.4. Growth Dynamics Curve

The growth dynamics curve was created following an established protocol [35]. Bacteria cultured overnight were diluted to approximately 1 × 10^6^ CFU/mL with sterile LB broth medium, and a certain concentration of QM-AgNPs was added to achieve final concentrations of 1/2MIC, 1MIC, and 2MIC for each bacterium. The same volume of PBS buffer was added to replace QM-AgNPs as a blank control. The absorbance of OD600 was measured every hour, and the growth dynamics curve of QM-AgNPs’ inhibition of bacteria was plotted.

#### 2.8.5. Antibiofilm

Biofilm is a three-dimensional structure formed by extracellular polymers (EPs) secreted by bacteria, containing polysaccharides, proteins, and nucleic acids, which is the external environment for bacteria. The determination of antibacterial film is based on the previously described method [36]. Biofilm formation was induced by adding bacterial suspension (1 × 10^6^ CFU/mL) to a 96-well plate and incubated at 37 °C for 48 h. The biofilm attached to the bottom of the wells was washed three times with PBS buffer. Different concentrations of QM-AgNPs were added to the wells, and the control group was incubated with culture medium for 24 h at 37 °C. The supernatant was discarded and the wells were washed with PBS buffer 2–3 times. Methanol was then added, and the biofilm was fixed for 30 min after discarding the methanol solution, and incubated for 15 min. The biofilm was washed 2–3 times with PBS buffer and 100 μL of 33% glacial acetic acid was added to each well for 15 min to decolorize the biofilm. The resulting supernatant was transferred to a new 96-well plate and the absorbance was measured at 570 nm using a microplate reader.

#### 2.8.6. Reactive Oxygen Species (ROS) Detection

Bacterial suspension at a concentration of 1 × 10^6^ CFU/mL was incubated with 2 × 10^−5^ mol/L 2′,7′-Dichlorofluorescin diacetate (DCFH-DA) in a constant-temperature incubator at 37 °C for 1 h. Excess extracellular DCFH-DA was removed by centrifugation at 4244× *g* for 10 min, and QM-AgNPs were added to achieve a final concentration of 1/2 MIC for each bacterium. Fluorescence intensity was measured using fluorescence spectrophotometry, with an excitation wavelength of 495 nm, an emission wavelength of 525 nm, and a slit width of 5 nm. This method is based on established oxidative stress detection protocols in bacteria [37].

#### 2.8.7. Measurement of Intracellular Contents

The quantification of nucleic acid and protein leakage was based on the previously described method [38]. Bacterial suspension at a concentration of 1 × 10^6^ CFU/mL was centrifuged at 10,611× *g* for 5 min to obtain the bacterial pellet. The supernatant was discarded, and the precipitate was washed 1–2 times with sterile PBS buffer. The bacterial pellet was resuspended in sterile PBS buffer and mixed with various concentrations of QM-AgNPs to achieve final concentrations of 1/2 MIC, MIC, and 2 MIC for each bacterium strain, followed by incubation at 37 °C for 4 h. The bacterial suspension was centrifuged at 10,611× *g* for 15 min every hour, and absorbance values at 260 nm and 280 nm were determined using a UV-visible spectrophotometer. (Sterile PBS buffer was used as a blank control in place of the QM-AgNPs solution).

#### 2.8.8. AO/PI Staining

Activated bacteria were treated with different concentrations of QM-AgNPs, achieving final concentrations of 1/2MIC, MIC, and 2MIC for each bacterial strain. Sterile PBS buffer was used as the blank control. The mixture was incubated at 37 °C for 4 h. After incubation, an AO/PI staining solution was added to the bacterial solution and incubated for 15 min in the dark. The staining solution was discarded and the wells were washed with sterile PBS buffer 2–3 times to remove excess dye and QM-AgNPs. Then, the remaining liquid was aspirated onto a slide, covered with a coverslip, and observed under a fluorescence inverted microscope. The staining results are visualized via fluorescence microscopy, following the method described previously [39].

### 2.9. Application in Grape Packaging

Coating grapes for freshness was performed according to the experimental method of Bizymis et al. [40,41,42,43]. A quantity of gelatin (GEL) was added to deionized water to achieve a concentration of 8% (W/V), to which a quantity of AgNPs solution was added and placed on a magnetic stirrer for dissolution and mixing. After mixing, the solution was defoamed using an ultrasonic apparatus. The prepared GEL and GEL@QM-AgNPs were stored at room temperature.

#### 2.9.1. Changes in Appearance 

We selected grapes of uniform shape, size, and ripeness; washed and dried them; and randomly divided them into three groups: control, GEL, and GEL@ QM-AgNPs. The control group was not treated; the remaining two groups were immersed in the coating solution of GEL and GEL@ QM-AgNPs for 10 s, after which they were removed to dry naturally. The grapes were stored under ventilated conditions at room temperature and photographed daily for observation. 

#### 2.9.2. Weight Loss

The weight loss of grapes was determined using the weighing method. The grapes were weighed daily and then evaluated according to the following formula:

Weight loss (%) = (M_0_ − M_1_)/M_0_ × 100%

In the formula, M_0_ represents the initial weight (g); M_1_ represents the weight after a certain period of time.

#### 2.9.3. Titratable Acidity

A total of 10 g of the sample was ground well and volumed to 50 mL with distilled water. A total of 0.1 mol/L of sodium hydroxide solution was titrated and a few drops of 1% phenolphthalein reagent were added until a slight red color developed and lasted for one minute without fading, repeating the process three times simultaneously. The formula was as follows:

The titratable acidity (%) = [C × (V − V_0_)]/[(V_2_/V_1_) × m] × 100%

where V is the volume of sodium hydroxide consumed in the titration of the sample (mL); V_0_ is the volume of sodium hydroxide consumed in the titration of the blank sample (mL); C is the concentration of the sodium hydroxide (mol/L); V_1_ is the raw volume; and V_2_ is the sample volume.

#### 2.9.4. Total Sugar Content

A certain amount of the grape was weighed, and a small amount of water was added to grind the pulp evenly; meanwhile, water was added to a constant volume of 250 mL. The solution was diluted 100 times after calibration, then a 2% anthrone solution was added to a boiling water bath for 10 min, and removed after cooling to room temperature at a 620 nm wavelength absorbance value, into glucose standard curve [24]. The calculation formula is as follows:

The total sugar content (mg/kg) = (W × M)/(N × 1000)

In this formula, W is the total soluble sugar content obtained by substituting the glucose standard curve (μg); M is the dilution factor; and N is the weight of the sample.

#### 2.9.5. Soluble Solids

The soluble solids content of the grapes was determined by squeezing the juice out of the grapes in each group and dropping the juice onto an Abbe refractometer.

#### 2.9.6. Browning Degree

A homogenate was prepared by mixing 2.0 g of sample with 20 mL of phosphate buffer solution with pH = 7.4, then centrifuged at 106× *g* for 5 min. The supernatant was obtained to measure the absorbance at 410 nm, and the value derived from A 410 × 10 was taken as the browning value.

#### 2.9.7. Spoilage Rate

The rate of decay was measured as the proportion of decay to the total number. The formula is as follows:

The spoilage rate (%) = (decaying fruit)/(total fruits) × 100

#### 2.9.8. Microbiological Evaluation

When the coating preservation experiment was carried out to the last day, the grapes were taken from each group and the surface of the fruits was rinsed with aseptic water. After rinsing, the water was added to Petri dishes and they were incubated at 37 °C for 16–20 h to observe the growth of the colonies. The colony growth was used to evaluate the preservation effect of AgNPs on grapes.

### 2.10. Statistical Analysis

All data were evaluated with three, parallel one-way Analysis of Variance (ANOVA) tests and expressed as mean ± standard deviation (SD). The significance variations were indicated at a threshold value of *p* < 0.05. All data were analyzed by origin Statistical and IBM SPSS Statistics Version 26 software programs.

## 3. Results

### 3.1. Optimization of Conditions for Green Synthesis

**Figure 1:**
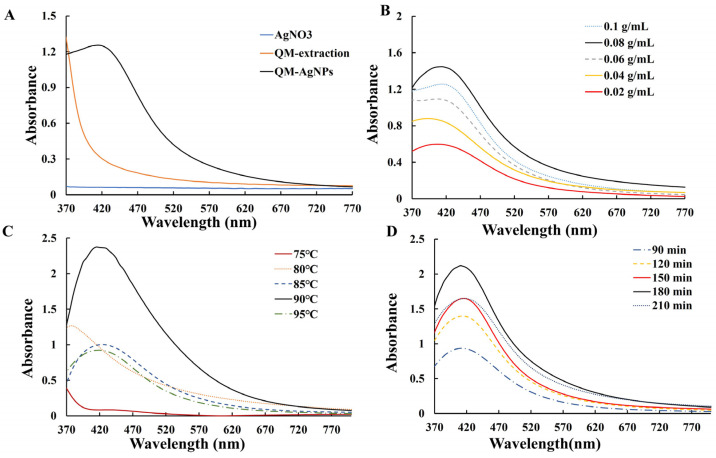
The UV-Vis absorption spectra of AgNO_3_, QM-extraction, and QM-AgNPs (**A**); UV-Vis absorption spectra of QM-AgNPs showing the effects of extract concentration (**B**); temperature (**C**); time (**D**).

Following the experimental methodology described above, the color changed to dark brown, indicating the successful synthesis of silver nanoparticles. Figure 1A shows the position of the characteristic absorption peak of QM-AgNPs. Figure 1B–D shows UV-vis spectra of silver nanoparticles synthesized under different conditions.

The UV-Vis spectra of AgNPs synthesized using QM extract at varying concentrations was illustrated. As seen in Figure 1B, with an increase in extract concentration, the number of bioactive ingredients in the system increases, accelerating the reaction rate and enhancing the absorption peak. The maximum absorption peak appeared at a concentration of 0.08 g/mL. When the concentration was increased to 0.1 g/mL, the absorption peak significantly decreased, likely due to the higher concentration of bioactive ingredients promoting the reduction of Ag^+^ and increasing the probability of agglomeration, thus reducing dispersibility and the absorption peak.

The ultraviolet spectra of AgNPs synthesized at different reaction temperatures are shown in Figure 1C. The absorption peak reached its maximum value at 90 °C, indicating that within certain limits, higher temperatures accelerated the reaction rate and promoted the synthesis of QM-AgNPs. However, further increasing the temperature caused a decrease in the absorption peak, likely due to the destruction of bioactive components in the extract at high temperatures, affecting the synthesis of QM-AgNPs.

Figure 1D depicts the UV-Vis spectra of AgNPs synthesized with an increase in synthesis time. The highest absorption peak was observed at 180 min, indicating the optimal reaction time for the maximum yield of QM-AgNPs. Extending the reaction time to 210 min resulted in a decrease in the absorption peak, possibly due to side reactions forming other substances that attached to the QM-AgNPs’ surface, leading to an increase in particle size and agglomeration, thereby reducing absorbance.

### 3.2. Analysis of Response Surface Results

**Figure 2:**
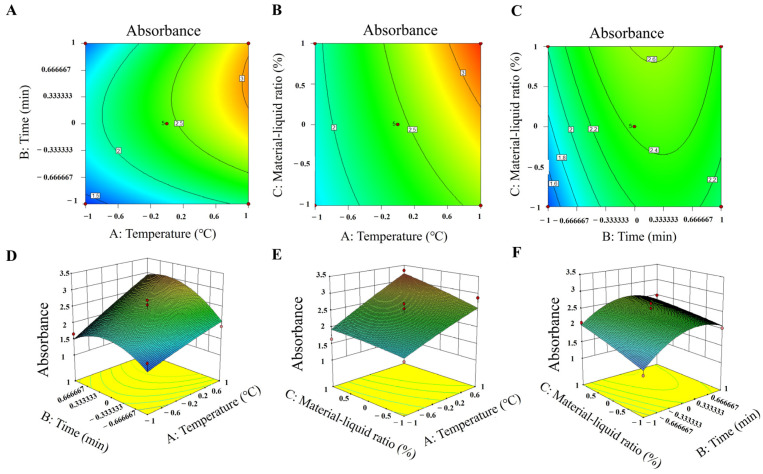
Response surface plots of the 2D contour and 3D surface plots of the QM-AgNPs. Temperature and time (**A**,**B**); extract concentration and temperature (**C**,**D**); extract concentration and time (**E**,**F**).

The experimental factors and response surface analysis are shown in Figure 2A–F and Tables S1 and S2. The 3D surface plots and contour plots indicated that the response surface initially increased and then decreased with increasing extract concentration and time. The interaction between extract concentration and time was significant, as indicated by the dense contour lines. Regrading the contours, the greater the inclination, the more significantly it affected the synthesis, so the concentration of the extract and the reaction time had a somewhat greater effect on the synthesis of QM-AgNPs. Error analysis and validation experiments confirmed the feasibility of the results, leading to the determination of the optimal conditions for synthesizing silver nanoparticles.

### 3.3. Characterization Analysis of QM-AgNPs

**Figure 3:**
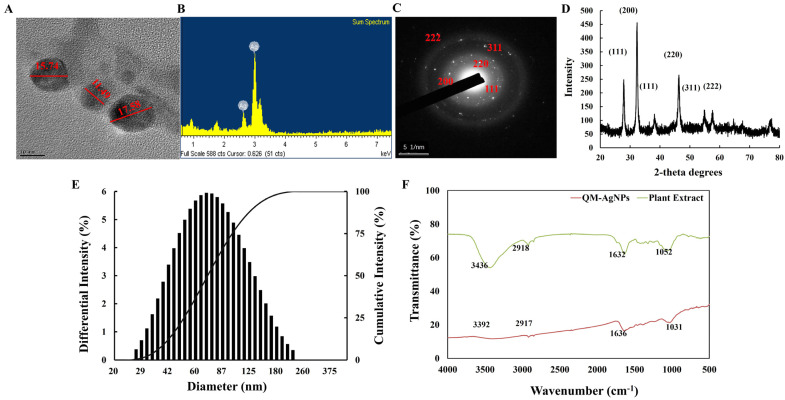
TEM images of spherical QM-AgNPs (**A**); EDX spectra of QM-AgNPs (**B**); XRD (**C**) and SAED (**D**) spectra of QM-AgNPs; particle size distributions of QM-AgNPs with respect to intensity (**E**); FTIR spectra of QM-AgNPs and QM power (**F**).

TEM is one of the important characterization methods to observe the morphology and particle size of QM-AgNPs. Figure 3A shows the electron microscopic image of QM-AgNPs with a spheroid shape, which is consistent with the previous research findings. Meanwhile, the particle size is mainly distributed between 11 and 18 nm.

EDS technology is instrumental in determining elemental composition. For QM-AgNPs, EDS analysis (Figure 3B) revealed a significant absorption peak corresponding to silver, indicating that silver is the primary component. This confirmed the successful synthesis of QM-AgNPs, verifying their elemental composition as predominantly silver.

SAED and XRD were utilized to analyze the phase composition and crystallinity of QM-AgNPs. As shown in Figure 3C,D, the presence of narrow peaks at multiple positions indicates the high crystallinity of the QM-AgNPs. The diffraction pattern displayed several strong peaks at angles corresponding to the (111), (200), (220), (311), and (222) planes of a face-centered cubic (fcc) silver structure. These findings are consistent with those reported in the literature [44,45,46].

The particle size of QM-AgNPs was measured by the dynamic light scattering technique (DLS). As shown in Figure 3E, the particle size distribution of QM-AgNPs’ hydrodynamics is close to a normal distribution. The results indicate that the synthesized QM-AgNPs have good dispersion. This may be due to the active compounds contained in the QM extract, which formed a protective layer around the particles with a spherical shape during the synthesis of AgNPs, ensuring a uniform shape and size. The particle size measured by TEM is the core size, which reflects the actual geometric diameter of silver nanoparticles. DLS measures the hydrodynamic diameter, which includes the core of the nanoparticles, the surrounding solvation layer, and the surface-bound biomolecules. Therefore, the particle size measured by DLS is larger than that measured by TEM.

Figure 3F displays the FTIR spectra of the plant extracts and QM-AgNPs. The plant extracts exhibited distinct absorption peaks at 3436 cm^−1^, 2918 cm^−1^, 1632 cm^−1^, and 1052 cm^−1^, corresponding to the stretching of O-H, polyphenols, asymmetric stretching of C-H, and S=O in carbohydrates, respectively. Similarly, the spectra of QM-AgNPs showed the stretching of O-H in carbohydrates at 3392 cm^−1^ and polyphenols at 2917 cm^−1^. The peaks at 1632 cm^−1^ and 1052 cm^−1^ in the plant extracts match with those at 1636 cm^−1^ and 1031 cm^−1^ in QM-AgNPs, indicating a crosslink between the plant extracts and AgNPs, which confirmed the effective coverage of organic molecules from the plant extracts on AgNPs [47]. Under optimal conditions, the metal ions immediately interacted with organic substances containing -OH and -COOH functional groups, altering protein conformation and facilitating the synthesis of AgNPs. The flavonoids, proteins, and water-soluble biomolecules in the plant extracts acted as reducing and stabilizing agents in the synthesis of QM-AgNPs.

### 3.4. Antioxidant Analysis of QM-AgNPs

**Figure 4:**
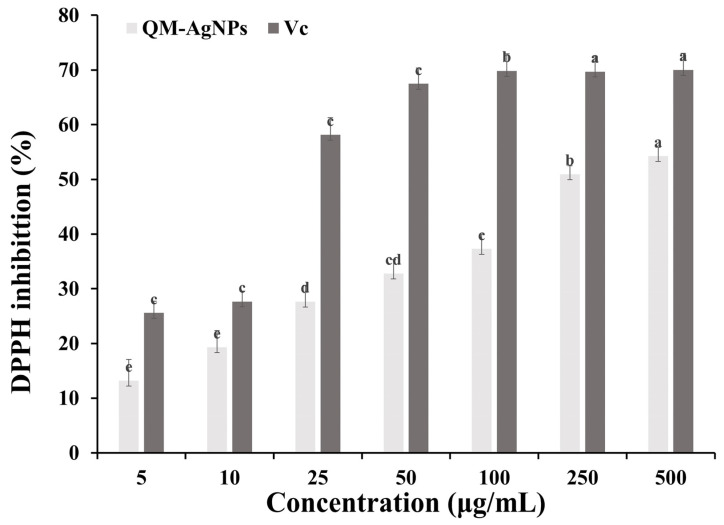
DPPH radical scavenging activity analysis of QM-AgNPs. Note: The DPPH radical scavenging effect of QM-AgNPs (5, 10, 25, 50, 100, 250, 500 μg/mL) was detected by DPPH radical scavenging assay; Vc (vitamin C) (5, 10, 25, 50, 100, 250, 500 μg/mL) was used as the positive control. Data are expressed as mean ± standard error (*n* = 3); different letters indicate significant differences (*p* < 0.05), based on one-way ANOVA followed by Tukey’s HSD test. (The analysis is focused on comparisons within the same group, that is, comparing the same sample at different concentrations).

It can be seen from Figure 4 that QM-AgNPs exhibit the significant ability of scavenging DPPH free radicals in a concentration-dependent manner. Since QM itself is rich in flavonoids, polysaccharides, and other active ingredients that can scavenge free radicals, when QM-AgNPs was synthesized from the QM extract, these active ingredients adhered to the surface of nanoparticles, promoted the formation of QM-AgNPs, and allowed it to possess an excellent free radical scavenging ability. This result is consistent with what has been previously reported in the literature [48,49,50,51].

### 3.5. Cytotoxicity Studies

#### 3.5.1. Cytotoxicity Evaluation

**Figure 5:**
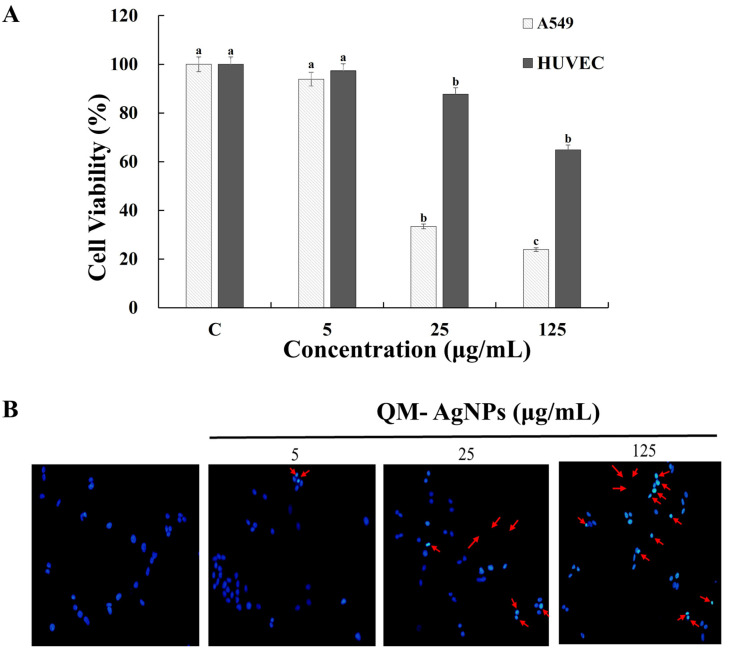
The results of the proliferation of QM-AgNPs (0, 5, 25, 125 μg/mL) on A549 and HUVECs (**A**); cell apoptosis of QM-AgNPs (0, 5, 25, 125 μg/mL) in lung cancer cells by Hoechst nuclear staining (**B**). Note: A549 and HUVECs were incubated with QM-AgNPs for 24 h. Cell viability was detected with MTT reagent. The arrows in the figure indicate apoptotic cells. Data are expressed as mean ± standard error (*n* = 3); different letters indicate significant differences (*p* < 0.05), based on one-way ANOVA followed by Tukey’s HSD test. (The analysis is focused on comparisons within the same group, that is, comparing the same sample at different concentrations).

In this study, cancer cells (A549) and normal cells (HUVECs) were selected as test subjects and treated with different concentrations of QM-AgNPs, and the results are shown in Figure 5A. Cell viability decreased in a dose-dependent manner with an increase in the QM-AgNPs concentration. These results indicate that QM-AgNPs shows anticancer activity against A549 cancer cells, while demonstrating no apparent cytotoxicity to HUVECs, with excellent biocompatibility. QM-AgNPs were able to damage cell components, causing functional defects and interfering with biological functions [52], and exhibited selective toxicity, strongly inhibiting tumor cells while reducing damage to normal cells.

#### 3.5.2. Cell Apoptosis Analysis

AgNPs have two main effects on cells: directly damaging the cell membrane and inducing reactive oxygen species (ROS) release, disrupting the cell’s molecular machinery through oxidative stress responses [53,54]. The apoptosis of lung cancer cells after treatment with different concentrations of QM-AgNPs was observed by Hoechst staining. As shown in Figure 5B, the nuclei of the control cells are larger and show a regular oval shape under the fluorescence microscope, whereas the cells treated with QM-AgNPs show apoptosis (the part of the cells highlighted by the arrows is whitish and glowing), and the number of apoptosis cells increases with the concentration. This may be due to the fact that AgNPs can induce cells to produce excessive reactive oxygen species (ROS), which leads to the damage of cellular organelles, such as mitochondria and lysosomes. In addition, AgNPs cause defective autophagy and inhibit mitochondrial autophagy, which ultimately activate the mitochondria-mediated apoptosis signaling pathway and cause cell death [55].

### 3.6. Antibacterial Activity of QM-AgNPs

#### 3.6.1. The Inhibition Zone

**Figure 6:**
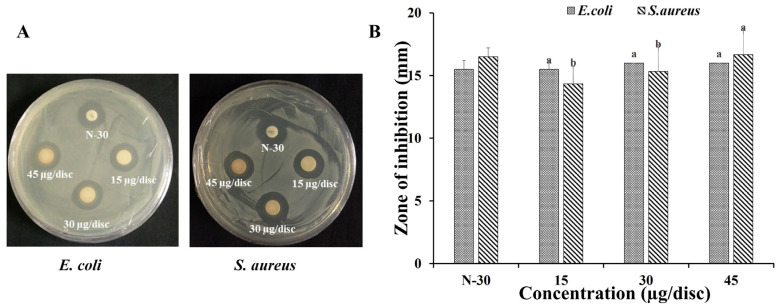
Zones of inhibition of N-30 and QM-AgNPs (15, 30, 45 μg/disc) against *E. coli* and *S. aureus* (**A**,**B**). Data are expressed as mean ± standard error (*n* = 3); different letters indicate significant differences (*p* < 0.05), based on one-way ANOVA followed by Tukey’s HSD test. (The analysis is focused on comparisons within the same group, that is, comparing the same sample at different concentrations).

The result of the inhibition zone is shown in Figure 6A,B, which can be concluded to increase with the increase in the concentration of AgNPs, indicating that the antibacterial effect of QM-AgNPs also improves with the increase in its concentration. Compared with the positive control, amikacin, QM-AgNPs showed similar antibacterial effects against both bacteria at the same concentration (30 μg/mL). However, it is not necessarily better with a higher concentration, and the dosage issue should also be considered [56].

#### 3.6.2. MIC and MBC

**Figure 7:**
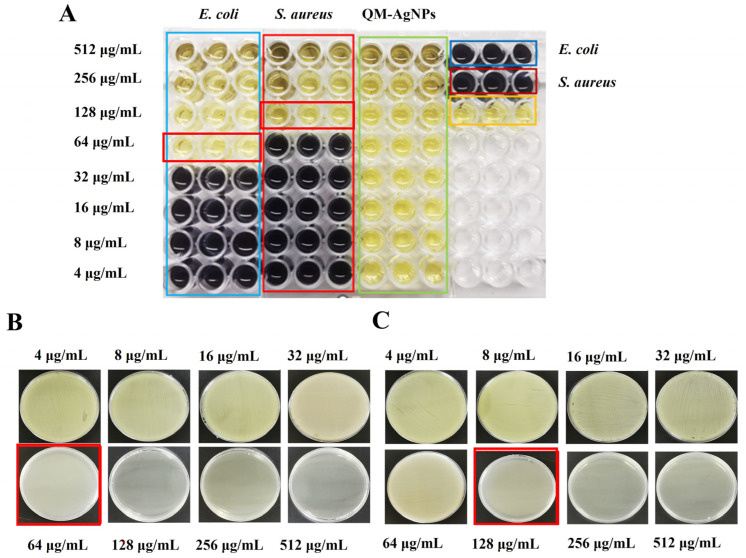
Analysis of MIC results against *E. coli* and *S. aureus* by QM-AgNPs (**A**). Analysis of MBC results against *E. coli* (**B**) and *S. aureus* (**C**) by QM-AgNPs. Note: The red boxes on the figure represent the MIC and MBC values of the corresponding bacteria. The boxes in other colors are only used to distinguish between different treatment groups.

The antibacterial results of QM-AgNPs measured by the 96-well plate method [57] are shown in Figure 7A–C. The MIC values of QM-AgNPs against *S. aureus* and *E. coli* were 32 μg/mL and 16 μg/mL, respectively, indicating that QM-AgNPs had outstanding resistance against both *S. aureus* and *E. coli*. The lower MIC value of *E. coli* reflected its higher sensitivity to QM-AgNPs compared to *S. aureus*. The main reason for the different resistance of the two bacteria is the difference in their cell structure [58]. There is no cell wall in Gram-negative bacteria and QM-AgNPs were more likely to enter the bacterial cell. The cell wall of Gram-positive bacteria is composed of thick peptidoglycan, and the peptidoglycan layer is composed of linear polysaccharides crosslinked with short peptides, which makes the penetration of QM-AgNPs more difficult. The results of MBC show that the inhibitory effect of QM-AgNPs on Gram-negative bacteria (64 μg/mL) is stronger than on Gram-positive bacteria (128 μg/mL).

#### 3.6.3. Growth Kinetic Analysis

**Figure 8:**
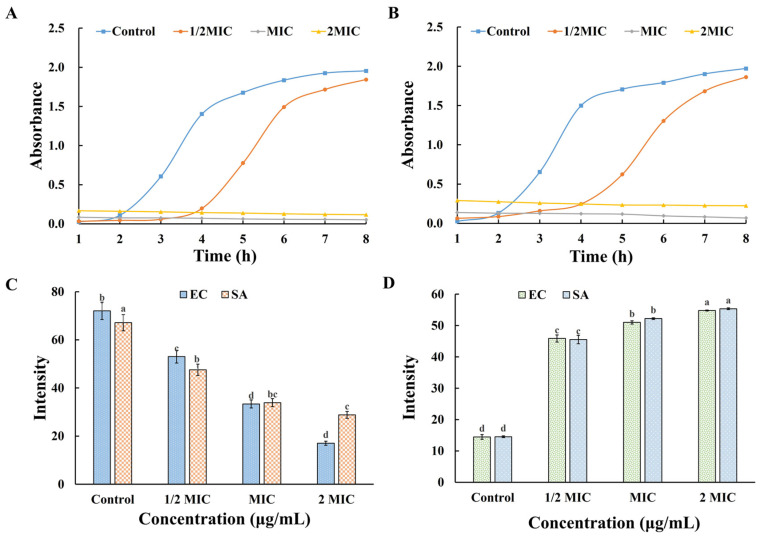
Growth curves of different bacteria under the action of QM-AgNPs, *E. coli* (**A**); *S. aureus* (**B**); anti-biofilm effects of QM-AgNPs (**C**); DFCH-DA measurement of ROS results (**D**). Data are expressed as mean ± standard error (*n* = 3); different letters indicate significant differences (*p* < 0.05), based on one-way ANOVA followed by Tukey’s HSD test. (The analysis is focused on comparisons within the same group, that is, comparing the same sample at different concentrations).

As shown in Figure 8A,B, in the blank control group, the growth kinetics of bacteria follows a typical pattern, including the lag phase, exponential phase, and stationary phase of bacterial growth, with the fastest growth rate in the first 4 h, after which the rate gradually decreases or even ceases, and finally reaches a plateau, and the growth curve shows an S-shape. In the presence of AgNPs, although the curves still conformed to the typical law, the growth of bacteria showed strong concentration dependence. When the concentration of AgNPs was lower than 1 × MIC, as the concentration of AgNPs increased, the growth of the two kinds of bacteria was delayed to a certain extent. When the concentration of QM-AgNPs reached MIC and 2MIC, the tested bacteria showed little signs of growth. The above results show that QM-AgNPs can inhibit the growth and reproduction of the tested bacteria, and demonstrate a dose-dependent effect. The reason for this phenomenon was mainly due to the fact that QM-AgNPs stimulated the bacteria, causing cell rupture, and thus led to the death of the bacteria. When the concentration of QM-AgNPs was relatively low, it had a certain inhibitory effect on the bacteria at the initial stage of action, but with the increase in the time of action, the reproduction rate of the bacteria became greater than the rate of action of the QM-AgNPs, which resulted in the rapid increase in the bacteria. And when the concentration of QM-AgNPs was high, all the bacteria present in the system could be killed in the reaction period, so the concentration of the bacterial solution would not increase with the increase in the action time.

#### 3.6.4. Antibiofilm Analysis

As shown in Figure 8C, after treatment with QM-AgNPs, the OD570 values of the four tested bacteria show a downward trend, and their biofilm clearance ability is enhanced with the increase in QM-AgNP concentration. When the concentration of QM-AgNPs reached 2MIC, the OD570 of the tested bacteria was much lower than that of the control group. The results show that QM-AgNPs have good biofilm clearance on both strains. When the concentration of QM-AgNPs was low, the inhibitory effect on bacteria was not obvious, and only a small amount of bacterial biofilm was destroyed, and it no longer had an effect after reaching a certain threshold. With an increase in the concentration of QM-AgNPs, all the bacteria present in the system could be destroyed, resulting in biofilm damage. This was due to the fact that as the QM-AgNP concentration increased, the stimulatory effect on the bacteria was more intense, leading to the destruction of more bacterial cells, which in turn caused the bacterial biofilm to rupture. 

#### 3.6.5. ROS

Previous studies [31,59,60] have shown that nano-based antimicrobial mechanisms are mostly related to inducing intracellular ROS production. According to previous studies, the pathways of AgNP-induced ROS can be summarized as follows: (1) In the extracellular environment, AgNP particles can induce the production of oxygen radicals (O·) and superoxide oxygen radicals (O_2_^−^·) during leaching, which are considered to be an important cause of cell membrane rupture; (2) at the cell membrane, AgNPs are likely to induce the leakage of electrons from the respiratory chain enzymes in the membrane into the cytoplasm and react with oxygen (O_2_) in the cytoplasm to form superoxide radicals (O_2_^−^·); and (3) intracellularly, the formation of superoxide radicals (O_2_^−^·) can destroy Fe-S clusters, release Fe^2+^, and participate in the Fenton reaction to reduce hydroxyl radicals (OH·). Ultimately, free radicals or ROS led to conformational changes in membrane proteins and DNA structure, ultimately leading to cell death. In this study, the relationship between the antibacterial activity of QM-AgNPs and ROS production was further investigated. The intracellular ROS production of bacteria treated with QM-AgNPs was detected using a DCFH-DA probe. As shown in Figure 8D, the fluorescence intensity of the tested bacteria without nano-treatment was low, indicating a low content of reactive oxygen species. After QM-AgNPstreatment, the fluorescence intensity increased sharply, indicating a surge in ROS content in the bacteria. The results show that the ROS content in *E. coli* increased further, due to the cell wall of Gram-negative bacteria being thinner, and QM-AgNPs being easier to enter, thus promoting the generation of reactive oxygen species and leading to the death of bacteria.

#### 3.6.6. Membrane Permeability Analysis

**Figure 9:**
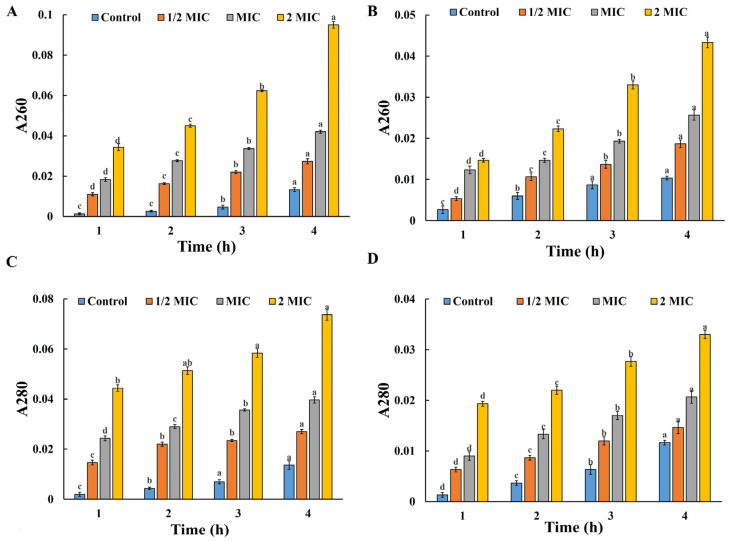
Effect of QM-AgNPs on extracellular nucleic acid leakage from different bacteria: *E. coli* (**A**); *S. aureus* (**B**). Effect of QM-AgNPs on extracellular nucleic acid leakage from different bacteria: *E. coli* (**C**); *S. aureus* (**D**). Data are expressed as mean ± standard error (*n* = 3); different letters indicate significant differences (*p* < 0.05), based on one-way ANOVA followed by Tukey’s HSD test. (The analysis is focused on comparisons within the same group, that is, comparing the same sample at different concentrations).

As described in other research [61,62,63], AgNPs can alter the permeability and respiratory characteristics of cell membranes, thereby inhibiting bacterial growth. When bacteria are damaged under the influence of nanoparticles, components such as proteins and nucleic acids leak out of the cell. As can be seen in Figure 9A–D, the OD260 nm and OD280 nm values of the blank control group, which were not treated with QM-AgNPs, did not change significantly during the reaction time, indicating that the bacterial membranes remained intact, and the bacteria maintained normal growth; whereas, under the treatment with QM-AgNPs, as the reaction time was prolonged, the OD260 nm and OD280 nm values increased gradually. This indicates that the bacterial intracellular substances progressively leaked out with the extension of the reaction time, indicating that bacterial growth was inhibited with the concentration-dependent effect. The increase in the leakage of bacterial intracellular substances indicates that the leakage of bacterial intracellular substances further emphasizes that this leakage was time-dependent. On the other hand, the leakage of bacterial intracellular substances was dose-dependent, which was consistent with the observed antibacterial activity of QM-AgNPs. Therefore, *E. coli* and *S. aureus* treated with QM-AgNPs exhibited membrane damage and the leakage of intracellular substances, which are important factors leading to bacterial death.

#### 3.6.7. AO/PI

**Figure 10:**
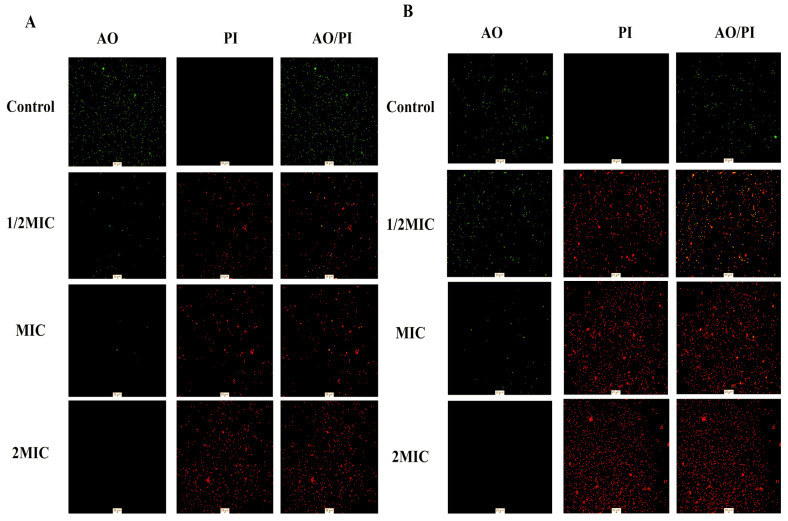
AO/PI staining results of QM-AgNPs acting on *E. coli* (**A**) and *S. aureus* (**B**).

To further confirm that QM-AgNPs can cause bacterial cell membrane rupture and lead to bacterial death, AO/PI fluorescence staining experiments were conducted [39]. Figure 10A,B shows that the bacteria treated without QM-AgNPs do not exhibit apoptosis and display green fluorescence. With the addition of QM-AgNPs, the bacterial cell membrane was disrupted, leading to bacterial death, which caused PI to stain the nucleus, resulting in red fluorescence. As the concentration of nanoparticles increased, more bacterial apoptosis occurred. Under the fluorescence microscope, most bacteria exhibited yellow–green or red fluorescence, which may be attributed to the binding of fluorescent dyes to nucleic acids in apoptotic or dead bacterial cells. When the concentration of QM-AgNPs reached 2MIC, all the bacteria died, and only red fluorescence appeared in the visual field. These results indicate that QM-AgNPs have a destructive effect on the bacterial cell membrane, leading to the deterioration of bacterial survival and ultimately causing bacterial death, which is consistent with the findings of previous studies [64,65].

**Figure 11:**
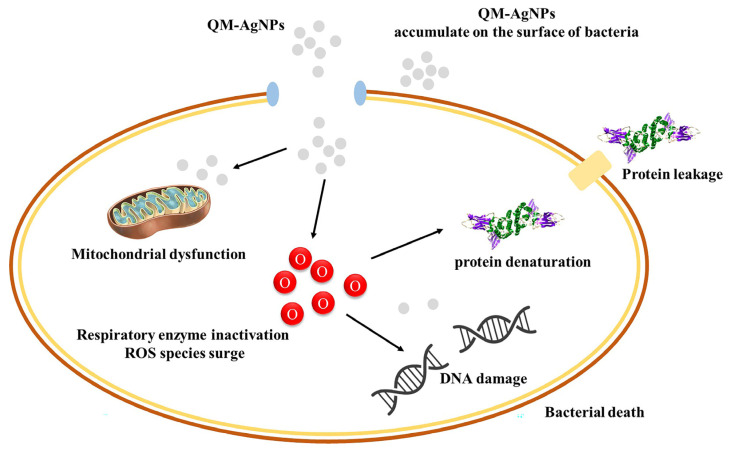
Mechanism of bacterial death caused by QM-AgNPs.

Based on the above studies of antibacterial mechanisms, we can briefly guess the mechanism by which QM-AgNPs cause bacterial rupture as follows (Figure 11): firstly, the nanoparticles will accumulate on the bacterial surface in large quantities, destroying the structural integrity of the cell membrane and leading to the leakage and denaturation of proteins in the cell; secondly, they can penetrate the bacteria to enter the intracellular space, directly damaging the DNA of the genetic material. What is more important is that the QM-AgNPs will attack the mitochondria in a targeted manner, inhibiting the activity of key enzymes of the respiratory chain. At the same time, QM-AgNPs induced a large accumulation of reactive oxygen species (ROS), resulting in oxidative stress damage. This multi-target, multi-level combined effect makes it difficult for bacteria to develop drug resistance, which ultimately leads to their death.

### 3.7. Fruit Preservation

#### 3.7.1. Appearance Changes Analysis

**Figure 12:**
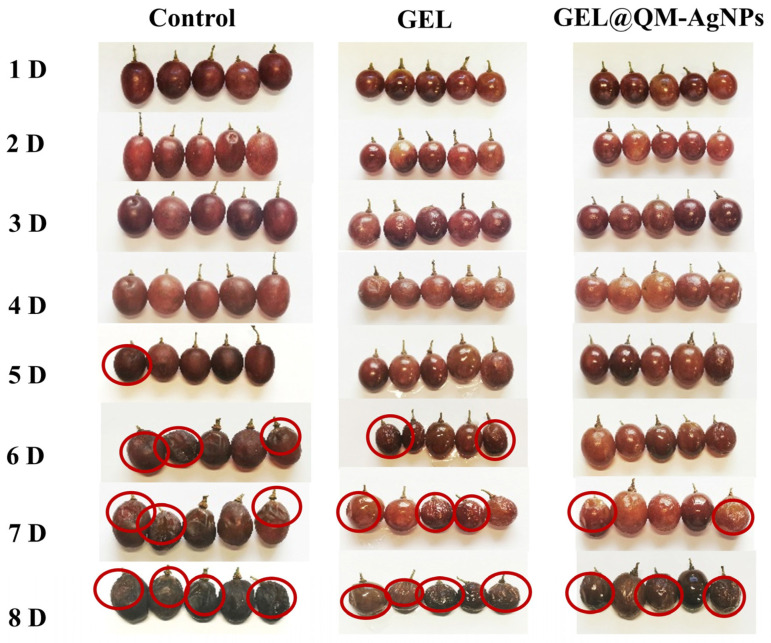
The changes in the appearance of different grape samples during storage. Note: The marks in the picture indicate where the grapes have rotted.

As shown in Figure 12, a certain degree of decay appeared on the fourth day in the blank group without any treatment. With the increase in time, the number of decayed grapes gradually increased, and even fruit flies and larvae appeared on the surface of grapes [66]. By the seventh day, most grapes exhibited severe decay with visible signs of spoilage, including mold growth and tissue maceration. The surface of the GEL-treated grape samples wrinkled to a certain extent due to water loss in the last few days, but no decay occurred, indicating that GEL could slow down the decay of the grapes to a certain extent. In the samples treated by GEL@QM-AgNPs, there were still more well-preserved grape samples on day 8, and no mildew spots appeared.

#### 3.7.2. Weight Loss Analysis

**Figure 13:**
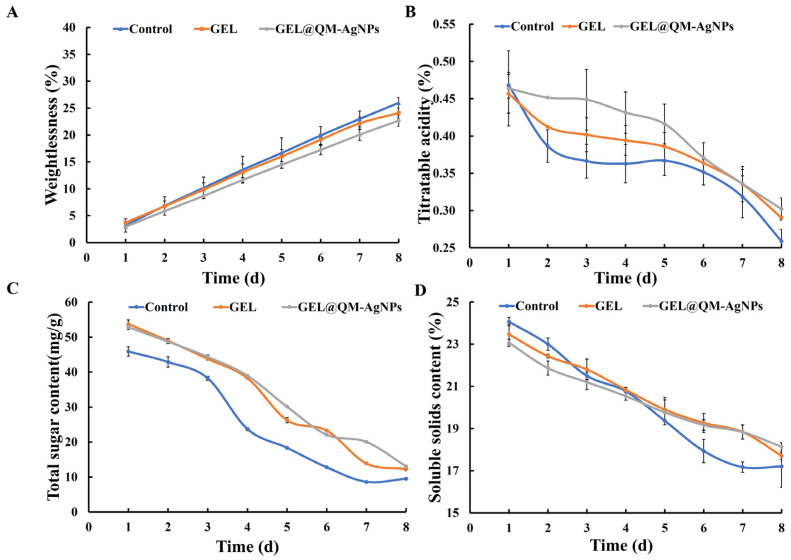
The weight loss of grapes in different types of film (**A**); changes in the titratable acidity of different types grapes (**B**); changes in the total sugar content of grape samples (**C**); changes in the soluble solids of grape samples (**D**).

Weight loss rate is an important parameter to measure the shape and quality of fruit during storage. Figure 13A shows the variation in the weight loss rate of grape samples under different treatment conditions. During storage, the weight loss rate of untreated grape samples was significantly higher than that of the other two groups, mainly due to the evaporation of water caused by the respiration and transpiration of the grapes during storage. The GEL group could not adhere to the grape’s surface because of its low adhesion, which could only reduce water loss to a certain extent. With the addition of AgNPs, the GEL@QM-AgNPs group altered the passage of water vapor, which reduced water loss to some extent [67]. The reason for this change in the weight of the grapes in the different groups is that the coating minimizes the contact of the grapes with the atmosphere and slows down the conversion of the pectin substances that maintain the structure of the cell walls to the non-adhesive pectic acid, which ultimately slows down the dehydration and wrinkling of the grapes. This phenomenon suggests that it is the barrier effect of the film coating that plays a dominant role in the apparent changes in film-coated grapes. In conclusion, the use of film coatings to preserve grapes seals the surface of the grapes and reduces their contact with the atmosphere, which in turn attenuates the degree of water loss and respiration.

#### 3.7.3. Analysis of Titratable Acidity

Titratable acidity measures the change in acidity of fruit during storage. As shown in Figure 13B, the titratable acidity of all grape samples decreased with storage time, resulting in a decrease in acidity between the blank control group and the coated group. The reason for the difference between the blank control group and the film-coated treatment group may be the respiration of the grapes in the blank control group was not hindered [68], so their titratable acids were converted into sugars at a faster rate. The difference in grapes between the film-coated treatment groups may be due to the different components of each film-coated treatment group: AgNPs in the GEL@QM-AgNP film have an antioxidant capacity, which can reduce the respiratory rate of grapes and delay the process of post-ripening in grapes. The results show that GEL@QM-AgNP coating on the fruit surface could inhibit the respiration of the fruit and reduce the consumption of organic acids.

#### 3.7.4. Total Sugar Content Analysis

Figure 13C reflects the changes in the total soluble sugar content in grapes under different treatment conditions. The total soluble sugar content of grape samples in the different treatment groups decreased. Sugar respiration in plants is transformed into other substances through glycolysis and other processes; the stronger the respiration, the more sugar and other substances are consumed, and the more energy is generated, making it difficult for fruits and vegetables to be preserved for a long time. Although other substances can be converted into glucose through gluconeogenesis and other pathways, the level of conversion was insufficient to offset consumption, resulting in the total sugar content showing a downward trend. Compared with the control group, the changes in total sugar content in the experimental group were significantly slowed down, indicating that the coating could inhibit respiration to a certain extent, thus slowing the decline in total sugar content. The GEL@QM-AgNP coating effectively maintains the total sugar content of grapes at a high level, thereby prolonging the storage time of grapes.

#### 3.7.5. Analysis of Soluble Solids

Figure 13D shows the change in the soluble solid content of grape samples in different treatment groups. The soluble solids of all grape samples showed a tendency to decrease. The glycogen conversion of grape samples during storage is insufficient to replenish soluble sugars and other substances consumed by respiration, resulting in the reduction in soluble solids [69]. The coating of the experimental group can effectively inhibit respiration and reduce the consumption of soluble sugars and other components in grape samples, thus slowing down the change in soluble solids and inhibiting the corruption of grape samples. The results show that the soluble solids in the GEL@QM-AgNPs group decreased the most slightly, indicating that the coating of this treatment group could effectively inhibit the respiration of fruits and prolong the storage life of fruits.

#### 3.7.6. Browning Analysis

**Figure 14:**
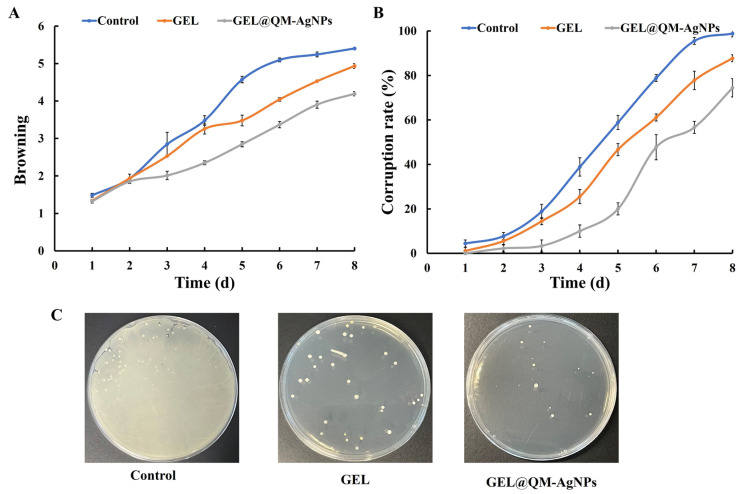
The changes in the browning degree of grape samples (**A**); changes in the corruption rate of grape samples (**B**); biological evaluation of the surface of grapes (**C**).

Browning degree is one of the criteria to evaluate the freshness of fruit. As can be seen from Figure 14A, with the prolongation of the storage time, the browning degree in the blank group is the greatest, and GEL@QM-AgNPs is the lowest. The reason may be that GEL@QM-AgNPs can greatly reduce the respiration of grape samples. The reduction of sugar consumption inside the fruit, as well as the addition of QM-AgNPs, can inhibit the microorganisms on the surface of the fruit, thus slowing down the browning of grape samples and extending the preservation time [70].

#### 3.7.7. Spoilage Rate Analysis

The spoilage rate is the most intuitive parameter to measure the freshness of fruit. Figure 14B shows the changes in the spoilage of grape samples in different treatment groups. The spoilage rate of all treatment groups increased with the storage time. When the grape samples were corrupted without any treatment, all of them were spoiled on the seventh day. The GEL group was able to form a coating on the surface of the grape samples, thereby reducing respiration and minimizing spoilage. However, due to its poor adhesion, the coating was separated from the grape epidermis as the grape samples lost water and shrank [71]. QM-AgNPs were added to the GEL@QM-AgNPs group to inhibit microorganisms on the grape’s surface, thus prolonging the storage time. The results show that the GEL@QM-AgNP coating can reduce respiration, inhibit microbial activity, and prolong the storage time of grapes to a certain extent.

#### 3.7.8. Microbiological Analysis

Figure 14C shows the microbial culture on the surface of different treatment groups on day 8. The number of bacterial colonies in the blank and GEL groups was significantly higher than that in the GEL@QM-AgNPs group, which was mainly due to the following reasons: firstly, the blank group lacked any antimicrobial components, providing an ideal growth environment for the microorganisms; and secondly, the simple GEL group, although it had a certain physical barrier effect, had limited antimicrobial activity, and was unable to effectively inhibit the reproduction of microorganisms. In contrast, the uniformly dispersed silver nanoparticles (QM-AgNPs) in the GEL@QM-AgNPs membrane were able to continuously release silver ions, and these silver ions significantly inhibited bacterial growth and reproduction by disrupting the microbial cell membranes, interfering with energy metabolism, and inducing oxidative stress, among other mechanisms [72]. Therefore, the total number of bacterial colonies in the AgNPs composite membrane group was lower, indicating that the GEL@QM-AgNPs membrane could greatly inhibit the growth and reproduction of microorganisms on the surface of the grapes, thus reducing the rotting of the grapes and prolonging the preservation time of the grapes.

The above experimental results demonstrate that the grapes treated with GEL@QM-AgNPs performed better than the other two groups. The possible reason for this is that the addition of QM-AgNPs reduced the pore area of the gel, stabilized the GEL@QM-AgNPs’ structure, and thus reduced the evaporation of water vapor and the diffusion of oxygen. At the same time, it weakened the respiration and transpiration of the fruit, reduced the consumption of nutrients in the fruit during storage, and achieved the purpose of extending the storage time. The effect of nano-silver on fruit preservation was verified.

## 4. Conclusions

Our study demonstrated that QM extract can be effectively used as both a reducing agent and a capping agent in the green synthesis of silver nanoparticles. SAED and XRD analyses confirmed that the QM-AgNPs possessed a cubic crystalline and metallic structure, while TEM analysis revealed their spherical morphology. FTIR analysis indicated that the active substances in the extract were successfully encapsulated around the silver nanoparticles. Cytotoxicity tests showed that QM-AgNPs had good biocompatibility and significant inhibitory effects on tumor cells. Additionally, the synthesized QM-AgNPs exhibited antibacterial activity against both Gram-positive *S. aureus* and Gram-negative *E. coli*. Furthermore, the coating treatment of grapes with QM-AgNPs extended their shelf life and reduced spoilage rates. This study provides a solid foundation for exploring the synthesis and biological applications of silver nanoparticles, contributing to the advancement of nanomaterial synthesis with broad development prospects.

## Figures and Tables

**Table 1:** Experimental factors and levels of the independent test variables.

Test Parameters	Levels of Test Parameters		
	−1	0	1
Temperature (°C)	85	90	95
Time (min)	150	180	210
Material–liquid ratio (%)	6	8	10

## Data Availability

The datasets generated for this study are available on request to the corresponding authors.

## Supplementary Materials

The following supporting information can be downloaded at: https://www.mdpi.com/article/10.3390/foods14132327/s1, Table S1: Response surface test design and results; Table S2: Analysis of variance of regression model.
