# Supplementary material for: Dianthus superbus L. (QM) Extract-Assisted Silver Nanoparticle Gelatin Films with Antioxidant and Antimicrobial Properties for Fresh Fruit Preservation
Source: Foods. 2025 Jun 30;14(13):2327. doi: 10.3390/foods14132327 (PMC12248836; doi:10.3390/foods14132327)
Supplement: Supplementary file 1 [file foods-14-02327-s001.zip › foods-3668576-supplementary.docx]

| Test run | Temperature（℃） | Time（min） | Material-liquid ratio（%） | Absorbance |
| --- | --- | --- | --- | --- |
| 1 | 0 | 1 | -1 | 2.5864 |
| 2 | 1 | 0 | -1 | 2.98267 |
| 3 | 0 | 0 | 0 | 2.41733 |
| 4 | 0 | 1 | 0 | 2.92667 |
| 5 | 0 | 1 | 1 | 2.50333 |
| 6 | -1 | 0 | 0 | 2.09 |
| 7 | -1 | 0 | 1 | 3.18833 |
| 8 | 1 | 0 | 0 | 2.54433 |
| 9 | 1 | 0 | 1 | 2.64367 |
| 10 | 0 | -1 | -1 | 2.34567 |
| 11 | 0 | 1 | 0 | 2.35133 |
| 12 | 0 | -1 | 0 | 2.30633 |
| 13 | 0 | -1 | 1 | 2.706 |
| 14 | 0 | 0 | 0 | 2.56833 |
| 15 | 1 | 0 | 0 | 2.37667 |
| 16 | -1 | -1 | 0 | 2.96 |
| 17 | -1 | 0 | -1 | 2.45333 |

**Table S1. Response surface test design and results**

**Table S2. Analysis of variance of regression model**

| Source | Sum of Square | Df | Mean square | F-value | P-value |  |
| --- | --- | --- | --- | --- | --- | --- |
| Model | 4.09 | 9 | 0.45 | 5.84 | 0.0148 | * |
| A-Temperature | 2.23 | 1 | 2.23 | 28.61 | 0.0011 | ** |
| B-Time | 5.00E-01 | 1 | 5.00E-01 | 6.37 | 0.0396 |  |
| C-Material-liquid ratio | 0.34 | 1 | 0.34 | 4.43 | 0.0733 |  |
| AB | 1.50E-01 | 1 | 1.50E-01 | 1.93E+00 | 0.207 |  |
| AC | 5.70E-02 | 1 | 5.70E-02 | 7.30E-01 | 0.4217 |  |
| BC | 0.025 | 1 | 0.025 | 0.32 | 0.5911 |  |
| A 2 | 3.30E-03 | 1 | 3.30E-03 | 0.042 | 0.8428 |  |
| B 2 | 7.70E-01 | 1 | 7.70E-01 | 9.89 | 0.0163 | * |
| C 2 | 3.35E-03 | 1 | 3.35E-03 | 0.043 | 0.8414 |  |
| Residual | 0.54 | 7 | 0.078 |  |  |  |
| Lack of Fit | 4.10E-01 | 3 | 1.40E-01 | 3.9 | 0.1106 |  |
| Pure Error | 0.14 | 4 | 0.035 |  |  |  |
| Cor Total | 4.63 | 16 |  |  |  |  |
| R^2^ | 0.8825 |  |  |  |  |  |
